# Dysfunctional high-density lipoprotein: an updated review

**DOI:** 10.3389/fcvm.2025.1713387

**Published:** 2025-12-16

**Authors:** Frances W. Ouyang, Huan-Hsing Chiang, Wen-Li Hsu, Ming-Hsien Tsai, Chun-Yao Huang, Alan T. Remaley, Omer Akyol, Chu-Huang Chen

**Affiliations:** 1 Molecular Cardiology Research Laboratories, Vascular and Medicinal Research, The Texas Heart Institute at Baylor College of Medicine, Houston, TX, United States; 2 National Center for Geriatrics and Welfare Research, National Health Research Institutes, Yunlin, Taiwan; 3 Department of Child Care, College of Humanities and Social Sciences, National Pingtung University of Science and Technology, Pingtung, Taiwan; 4 Division of Cardiology, Department of Internal Medicine, School of Medicine, Taipei Medical University, Taipei, Taiwan; 5 Lipoprotein Metabolism Laboratory, Translational Vascular Medicine Branch, National Heart, Lung, and Blood Institute, National Institutes of Health, Bethesda, MD, United States

**Keywords:** dysfunctional, high-density lipoprotein (HDL), electronegativity, cardiovascular disease, inflammation, oxidative stress

## Abstract

High-density lipoprotein (HDL) has earned its reputation as “good” cholesterol in cardiovascular health, primarily because of its strong inverse association with cardiovascular disease. A potential mechanism for this association is its ability to promote cholesterol efflux capacity (CEC) and consequently reduce the buildup of cholesterol in arterial plaque. However, recent research underscores the importance of not only maintaining high HDL cholesterol (HDL-C) levels but also ensuring the functionality and quality of HDL particles. HDL particles exhibit various other atheroprotective activities, including anti-inflammatory, antioxidant, and vasodilatory properties. Collectively, these functions are thought to contribute to reducing cardiovascular risk beyond mere cholesterol transport. Both acute and chronic inflammation can induce structural and functional changes in HDL, potentially rendering the particles pro-inflammatory. Factors that increase inflammation, such as lifestyle choices, autoimmune diseases, and oxidative stress, can adversely affect HDL functionality. Dysfunctional HDL, such as electronegative HDL H5 or HDL isolated from patients with cardiovascular disease (CVD), may lose its protective properties and even contribute to CVD progression by promoting inflammation, oxidative stress, and endothelial damage. Recent studies indicate that the CEC of HDL particles may serve as a more critical determinant of atheroprotection than the absolute concentration of HDL-C. This review emphasizes the need to focus on both quantity and quality of HDL to reduce cardiovascular risk more effectively. Understanding the mechanisms behind HDL's protective effects provide valuable insights into heart health and potential therapeutic strategies.

## 1 Introduction

### 1.1 Overview of HDL in cardiovascular health

High-density lipoprotein (HDL) has been widely regarded as a guardian against cardiovascular disease (CVD), fostering the assumption “the higher, the better”. However, emerging evidence reveals a paradox: not all HDL is inherently health beneficial. Recent findings from the Copenhagen study challenges the traditional narrative by revealing a U-shaped relationship between HDL cholesterol (HDL-C) levels and all-cause mortality, whereby both extremely low and excessively high HDL concentrations are linked to increased mortality, prompting a reexamination of HDL's role in human health (1).

Fundamentally, HDL is a complex lipoprotein, composed of many different types of lipids and proteins, with a high density (>1.063 g/mL) and small size (5–17 nm) (2). Its major protein is Apolipoprotein A-I (ApoA-I), accounting for approximately 60% of its total protein content, with ApoA-II contributing 20%, alongside other less abundant apolipoproteins and enzymes. Structurally, HDL contains an outer layer of free cholesterol, phospholipids, and apolipoproteins, while its hydrophobic core contains triglycerides (TG) and cholesteryl esters (CE) (3). Functionally, HDL plays a central role in reverse cholesterol transport (RCT), a process largely driven by ApoA-I, which facilitates cholesterol removal from peripheral tissues and its excretion by the liver (4). This process is thought to reduce atherosclerosis risk and protect against CVD. Cholesterol efflux capacity (CEC) is often considered a functional marker of HDL efficiency, reflecting its ability to facilitate cholesterol removal from peripheral cells. Beyond lipid transport, HDL also possesses anti-inflammatory, antioxidant, and endothelial-protective properties, which may help mitigate oxidative stress, reduce vascular inflammation, and promote arterial health (5).

It is important to note that HDL is not always universally beneficial. When HDL becomes dysfunctional through structural and functional changes, its CEC is impaired, diminishing its anti-inflammatory and antioxidant properties. Dysfunctional HDL may even promote pro-inflammatory and pro-thrombotic activities, increasing the risk of CVD (6). This new view challenges long-held assumptions about HDL and underscores the need to focus on its functionality rather than simply the level of cholesterol on HDL.

### 1.2 Rationale for focusing on dysfunctional HDL

While HDL has long been recognized for its numerous potential atheroprotective properties (Table 1), such as mediating RCT, exerting anti-inflammatory and antioxidative effects, and supporting endothelial health, recent studies reveal that HDL can lose these protective functions under certain pathological conditions, such as systemic inflammation and oxidative stress (7). In these states, HDL particles may undergo structural and compositional modifications, including oxidation and glycation, rendering them not only ineffective but potentially pro-inflammatory and pro-atherogenic (8). This functional impairment has been implicated in the promotion of atherosclerosis and CVD, even in individuals with normal or elevated HDL-C levels (9).

**Table 1:** A comparative profile highlighting the structural, molecular, and functional features that distinguish the atheroprotective H1 subfraction from the dysfunctional, pro-atherogenic H5 subfraction.

Features	H1 (Atheroprotective HDL subfraction)	H5 (Dysfunctional HDL subfraction)
Atheroprotective role	Predominantly protective	Pro-atherogenic; contributes to early atherosclerosis
Cholesterol efflux capacity (CEC)	High CEC; supports reverse cholesterol transport (RCT)	Reduced CEC due to structural and protein modifications
Electrophoretic mobility	Least electronegative; migrates farthest from anode	Most electronegative; migrates closest to anode
Size & density	Smaller, denser particles	Larger, less dense and heterogeneous
Antioxidant content	Lower overall antioxidant protein levels	Enriched in antioxidant proteins, but often oxidatively modified
Inflammatory profile	Anti-inflammatory	Pro-inflammatory; increases cytokine release (e.g., IL-1β, TNF-α)
Foam cell formation	Not typically associated	Promotes macrophage foam cell formation
Apolipoprotein composition	Enriched in native ApoA-I; low levels of ApoC-III, ApoE, SAA-1	High in oxidized ApoA-I, ApoE, ApoC-III
ApoA-I and ApoE integrity	Maintains native, functional structure	Susceptible to oxidative modification, disrupting lipid handling and promoting inflammation
Lipoprotein enrichment	Minimal abnormal lipoprotein accumulation	Elevated levels of modified lipoproteins and Lp(a)
Receptor interactions	Effective interaction with ABCA1 and SR-BI for cholesterol trafficking	Impaired receptor interaction: may involve scavenger receptors or contribute to dysfunction
Systemic effects	Supports vascular homeostasis and RCT	Contributes to oxidative stress, systemic inflammation, and lipid dysregulation
Percentage amount	High (average 83.8%)	Low (average 4.0%)

The clinical significance of dysfunctional HDL is emphasized by findings that simply raising HDL-C levels pharmacologically does not consistently translate into reduced cardiovascular events, as demonstrated in several large clinical trials of HDL-raising agents (9). This disconnect suggests that the qualitative aspects of HDL are as important, if not more so, than its concentration in plasma (10). Moreover, the development of assays to assess HDL functionality and the identification of biomarkers specific to dysfunctional HDL hold promise for refining cardiovascular risk assessment and guiding targeted therapies (11). By focusing on dysfunctional HDL, researchers and clinicians can better understand the complex role of HDL in cardiovascular health and disease, which may lead to more effective diagnostic and therapeutic strategies (11).

## 2 The evolving concept of dysfunctional HDL

### 2.1 Functional vs. dysfunctional HDL

**Figure 1:**
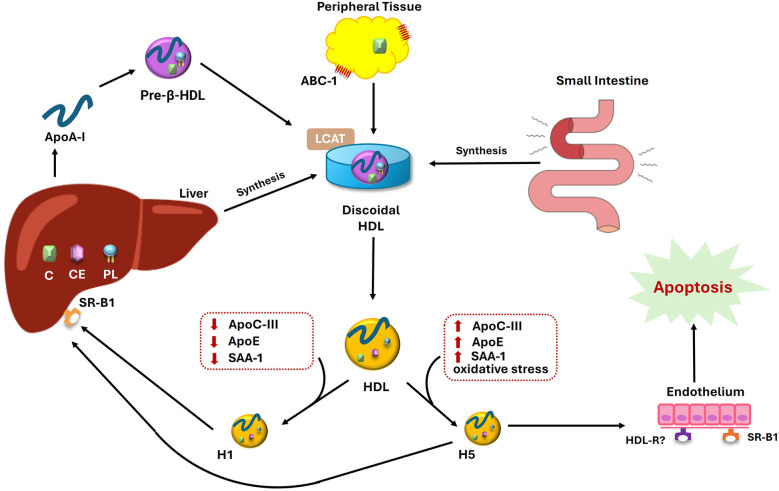
Formation of HDL subfractions and the pathogenic role of H5 in endothelial apoptosis. ApoA-I secreted by the liver and intestine initiates the formation of pre-β HDL, which acquires cholesterol from peripheral tissues via ABC-1 and matures into discoidal HDL. LCAT activity converts discoidal HDL into spherical HDL, which undergoes further remodeling into functionally distinct subfractions of HDL, H1 and H5. H1, with low levels of ApoC-III, ApoE, and SAA-1, is considered protective. In contrast, H5, enriched in these proteins and oxidative stress markers, promotes endothelial apoptosis and dysfunction.

Dysfunctional HDL is a modified form of HDL that loses its protective cardiovascular properties and instead contributes to inflammation, thrombosis, and other atherogenic processes (12). This dysfunction is driven by the incorporation of proinflammatory and prothrombotic proteins, such as Apolipoprotein C-III (ApoC-III), lipoprotein-associated phospholipase A2 (Lp-PLA2), and serum amyloid A1 (SAA-1) (Figure 1), which disrupts its normal functions (12).

The generation of dysfunctional HDL begins with the formation of nascent HDL particles, primarily in the liver and intestine (Figure 1). These lipid-poor particles consist mainly of ApoA-I and phospholipids and are initially functional, thereby facilitating RCT (13). However, during chronic inflammation and oxidative stress, and likely other unidentified reasons, HDL undergoes structural changes that impair its ability to interact with cholesterol transporters, thereby reducing its CEC (13). For instance, oxidative modifications to ApoA-I not only disrupt lipid metabolism but also shift HDL toward a pro-inflammatory profile. This profile is characterized by the upregulation of vascular adhesion molecules, enhanced monocyte recruitment, and foam cell formation (14, 15).

Furthermore, HDL particles enriched with ApoC-III exhibit reduced clearance from circulation and are strongly associated with insulin resistance, type 2 diabetes, and coronary heart disease (16). These alterations, including the increased concentration of ApoA-I within dysfunctional HDL, further differentiate it from its protective counterparts (16). Beyond lipid metabolism, HDL also plays a significant role in the innate immune system, contributing to pathogen neutralization and antiviral defense (17). However, the shift in HDL composition during chronic inflammation compromises its immune functions, exacerbating vascular dysfunction and promoting a pro-atherogenic state.

Specifically, the most electronegative subclass of HDL (H5) plays a critical role in the formation of foam cells (Table 1) by significantly reducing CEC due to protein modifications (18). H5 is the most electronegative and antioxidant-rich subclass, yet it paradoxically promotes inflammation by elevating levels of proinflammatory cytokines, including IL-1β and IL-8 (18). Moreover, the increase in H5 correlates with heightened inflammation, and its ApoC-III-rich composition further exacerbates these pathological effects (Figure 1).

### 2.2 Classification, subtypes, and underlying mechanisms of dysfunctional HDL

Dysfunctional HDL can be classified based on the nature of the modification or underlying mechanism. Dysfunctional HDL is commonly found in conditions of chronic inflammation, oxidative stress, diabetes, chronic kidney disease, and CVD (6). Its identification is clinically important, as dysfunctional HDL may not only fail to protect against atherosclerosis but may actively contribute to disease progression (6).

One key mechanism involves oxidative modifications of the major protein component of HDL, ApoA-I. In inflammatory conditions, enzymes such as myeloperoxidase (MPO) can oxidize ApoA-I, inducing conformational changes that impair its ability to promote cholesterol efflux from cells. This oxidation can also reduce HDL's interaction with cholesterol transporters like ATP-binding cassette transporter A1 (ABCA1) and ATP-binding cassette transporter G1 **(**ABCG1), further hindering RCT (19).

Glycation, the non-enzymatic binding of sugars to proteins, is another important mechanism, particularly in individuals with diabetes (20). Glycation of ApoA-I can alter its structure and lipid-binding capacity, reducing its ability to mediate cholesterol efflux. Glycated HDL may also have impaired antioxidant and anti-inflammatory functions (20).

Inflammation itself plays a significant role in the formation of dysfunctional HDL. During inflammatory responses, acute phase proteins like serum amyloid A (SAA) can displace ApoA-I on HDL particles (21). This altered protein composition can diminish HDL's antioxidant and anti-inflammatory capacities even rendering it pro-inflammatory. Additionally, inflammatory cytokines can affect the expression and activity of enzymes associated with HDL, such as paraoxonase-1 (PON1), which normally protects against LDL oxidation (22). Acute-phase HDL is produced after incorporation of acute-phase reactants like SAA during inflammation, which displaces ApoA-I and impairs HDL function (23).

Furthermore, changes in the lipid composition of HDL can contribute to dysfunction (24). HDL can acquire oxidized lipids or undergo *in situ* lipid oxidation, which can interfere with its antioxidant, anti-inflammatory, and cholesterol acceptor capabilities (25). An increased content of TG and oxidized phospholipids in HDL has also been observed in dysfunctional HDL (6). TG-rich HDL is a modified form of HDL commonly associated with metabolic syndrome or diabetes, which also alters its structure and diminishes its protective functions (23).

Alterations in the protein composition other than ApoA-I represent another mechanism contributing to the formation of dysfunctional HDL. Changes in the protein cargo of HDL, such as increased ApoC-I or decreased PON1, affects its antioxidant and anti-inflammatory properties (26).

Genetic factors can also predispose individuals to HDL dysfunction. Variants in genes encoding proteins involved in HDL metabolism, such as scavenger receptor class B type 1 (SR-B1), have been associated with altered HDL function and increased CVD risk (27).

## 3 Electronegativity and other physicochemical properties of HDL

To better understand the mechanisms underlying HDL dysfunction, it is essential to consider how variations in HDL electronegativity influence its biological activities. HDL subclasses can be categorized based on their electronegativity. Electrophoretic analysis of H1 through H5 shows that H5, which migrates closest to the anode, is the most negatively charged subclass (Table 1). While a greater negative charge generally enhances an HDL subclass's ability to promote cholesterol efflux, H5 is an exception. It is less effective in its cholesterol efflux abilities, likely due to its altered protein composition and impaired interaction with cholesterol transporters (3).

The electronegativity of HDL is primarily determined by its protein components, particularly apolipoproteins, such as ApoC-III (Figure 1), which carry strong negative charges due to their sialic acid residues (3, 28). The higher the concentration of apolipoproteins, the more negatively charged the HDL particle becomes, as apolipoproteins tend to have a low isoelectric point (pI) (29). The overall negative charge of HDL is crucial for its interactions with cell membranes, receptors, and enzymes, which are integral to its role in lipid metabolism and RCT. Additionally, oxidative modifications that increase the negative charge of ApoA-I, along with the replacement of ApoA-I by SAA-1 during oxidative stress, further enhance the overall negative charge of HDL particles (3).

The surface of HDL particles is composed of a monolayer of phospholipids and apolipoproteins, along with free cholesterol. The phospholipids, particularly phosphatidylserine, contribute significantly to the net negative charge of the HDL particle at physiological pH (30). This negative charge is not just a static property; it is dynamic and can be influenced by the lipid and protein composition of HDL (3), which can change in various physiological and pathological states.

The negative charge on HDL can alter electrostatic interactions with the generally negatively charged surfaces of cell membranes. Although this interaction might involve more complex mechanisms than simple electrostatic attraction, the charge can influence the proximity and orientation of HDL particles relative to the cell surface (31). HDL interacts with several receptors, most notably SR-B1 on liver and steroidogenic cells (32). While the primary interaction with SR-B1 involves hydrophobic interactions with lipids, the overall charge distribution on HDL can influence its binding affinity and selectivity for these receptors, affecting the selective uptake of CE from HDL (33). Changes in HDL charge, such as those occurring with modifications like oxidation or glycation, can alter its interaction with SR-B1, impacting cholesterol delivery to the liver.

Enzymes like lecithin-cholesterol acyltransferase (LCAT) and cholesteryl ester transfer protein (CETP) are key players in HDL metabolism and RCT (34). LCAT on HDL esterifies free cholesterol into CE, which then moves into the core of the HDL particle, increasing its capacity to accept more free cholesterol. CETP mediates the exchange of CE from HDL for TG from TG-rich lipoproteins. The activity of these enzymes can be influenced by the surface properties of HDL, including its charge. For instance, changes in the phospholipid composition and the presentation of ApoA-I due to alterations in charge distribution are known to alter LCAT activity (35). Together, these charge-dependent alterations underscore how deviations in HDL electronegativity contribute directly to its transition from a protective to a dysfunctional particle.

## 4 Methods for assessing HDL quantity and function

### 4.1 HDL-C measurement: strengths and limitations

HDL-C quantification is a cornerstone of cardiovascular risk assessment, primarily due to its well-established inverse association with atherosclerotic CVD (ASCVD) from numerous large scale epidemiologic studies (36). The most widely used method for HDL-C measurement are homogeneous assays, which employ wide varieties of approaches, such as the selective detergent-based solubilization of non-HDL lipoproteins, followed by enzymatic cholesterol measurement. These assays are highly automated, cost-effective and well standardized by the CDC's Cholesterol Reference Method Laboratory Network (CRMLN) (37) and correlate well with gold-standard ultracentrifugation approach (38). However, a key limitation is their inability to distinguish between HDL subclasses (HDL2 and HDL3), which may have divergent functional and atheroprotective properties (39). Additionally, homogeneous assays can lose their specificity for measuring only cholesterol on HDL with dyslipidemias and other conditions (40, 41).

Despite its widespread use as a routine diagnostic test, HDL-C concentration alone does not fully capture HDL's functional capacity. For example, HDL-C levels do not closely correlate with its CEC as measured by the ‘CEC assay’, critical metric of HDL functionality strongly linked to ASCVD risk independent of HDL-C levels (5). Studies suggest that HDL particle number (HDL-P), measured via nuclear magnetic resonance (NMR) spectroscopy, may better predict cardiovascular outcomes than HDL-C (42). Furthermore, inflammatory states such as acute-phase responses can render HDL dysfunctional, impairing its atheroprotective roles even when HDL-C levels appear normal (43). Thus, while HDL-C remains a key biomarker in risk stratification, emerging data emphasize the need for complementary metrics, such as HDL function assays or advanced lipoprotein profiling, to refine cardiovascular risk prediction (44).

### 4.2 Emerging techniques for characterizing HDL subfractions

Recent advances in lipoprotein analytics have enabled more precise characterization of HDL subfractions, which vary in size, density, and functionality. Assessment of HDL-P and subclass composition via NMR offers a more informative measure of cardiovascular risk than conventional HDL-C quantification (26, 42, 45). Ion mobility spectrometry (IMS) provides high-resolution separation of HDL subspecies based on electrophoretic mobility, revealing distinct associations with coronary artery disease (CAD) (46). Additionally, mass spectrometry (MS)-based proteomics and lipidomics have uncovered compositional differences in HDL subpopulations linked to atheroprotection or dysfunction (47). Although these different methods improve our mechanistic understanding, issues with standardization and higher costs have restricted their widespread use in routine clinical practice. Future refinements may better integrate these techniques for personalized HDL function assessment.

Assessing dysfunctional HDL requires a combination of biochemical, immunological, and functional assays to characterize changes in its composition and activity. Immunoseparation techniques differentiate HDL subtypes based on their apolipoprotein content, distinguishing between particles that contain only ApoA-I and those that also include ApoA-II (48). Since ApoA-I is considered one of the most important atheroprotective components of HDL, its relative abundance may provide important insight into HDL function (49).

Inflammatory profiling techniques serve as key tools for evaluating HDL functionality, indicating whether it maintains its anti-inflammatory actions such as inhibiting LDL oxidation and limiting monocyte recruitment to the arterial wall. Systemic inflammation, oxidative stress, and metabolic conditions like CVD and diabetes can induce structural and compositional changes in HDL, converting it from an anti-inflammatory to a pro-inflammatory particle. Functional impairment can be evaluated through assays that quantify HDL's ability to counteract LDL-induced monocyte chemotactic activity in artery wall coculture models, effectively reflecting its anti-inflammatory capacity (12).

### 4.3 Advanced assays: CEC, antioxidative function, and proteomics

Another widely used method for evaluating HDL function is the CEC assay, which measures HDL's ability to remove cholesterol from cells in tissue culture like macrophages, a crucial step in RCT (5). MS-based proteomic and lipidomic assays precisely detect dysfunctional HDL by identifying oxidative modifications of ApoA-I that impair CEC (50). These assays provide direct evidence of HDL's role in cholesterol metabolism and highlight potential dysfunction in disease states.

Together, these methodologies offer a comprehensive approach to distinguishing between protective and dysfunctional HDL subtypes. Understanding these differences is essential for elucidating HDL's role in atherosclerosis progression and cardiovascular risk.

## 5 Determinants of HDL level and function

### 5.1 Genetic and epigenetic factors

HDL-C levels are influenced by a combination of genetic, lifestyle, metabolic, and environmental factors. Genetic predisposition accounts for 40%–60% of HDL-C variability, with both monogenic disorders like familial hypoalphalipoproteinemia and polygenic factors (26, 51).

Genetic variations that affect apolipoprotein E (ApoE) receptor binding play a critical role in modulating HDL levels and composition, with implications for lipid metabolism and cardiovascular risk (52). However, HDL is also shaped by disease-related changes, particularly in chronic inflammatory conditions. In autoimmune diseases such as rheumatoid arthritis (RA) and systemic lupus erythematosus (SLE), HDL levels are influenced not only by genetic predisposition but also by systemic inflammation, altered lipid metabolism, and immune dysregulation (53). Understanding the interplay between genetic and inflammatory factors is essential for evaluating cardiovascular risk and disease activity in these patients. This multifactorial perspective sets the stage for exploring the broader role of HDL, particularly the H5 subclass, in CVD.

ApoE is a 34 kDa polymorphic glycoprotein primarily secreted by the liver but is also produced by other cells like macrophages in atherosclerotic lesions (54). Genetic variations in ApoE affect its interaction with lipoprotein receptors like LDL receptor (LDLR), influencing lipid clearance and HDL composition. ApoE isoforms exhibit distinct effects on lipid metabolism. ApoE2 has reduced affinity for LDLR, leading to impaired remnant clearance and lipid accumulation, while ApoE4 binds more avidly and alters lipoprotein cargo, promoting lipid peroxidation, but is associated with elevated LDL-C levels, and disruption of HDL metabolism. In contrast, the rare ApoE3 Christchurch variant reduces LDLR binding similar to ApoE2, but it may confer protection against pathological lipid oxidation. In mice, which express only a single ApoE isoform, dominant mutations primarily cause hypertriglyceridemia, whereas in humans they can result in disorders such as type III hyperlipoproteinemia (55–57). Homozygous ApoE deficiency is linked to a pro-atherogenic lipoprotein profile and heightened atherosclerotic risk. Like ApoA-I, ApoE also facilitates cholesterol efflux, preventing foam cell formation (58–60), but certain variants, such as ApoE4, promote dysfunctional HDL subtypes by increasing TG-rich lipoproteins and impairing cholesterol transport. Additionally, HDL-associated ApoE may become dysfunctional when it contains ApoC-III, which inhibits lipoprotein lipase (LPL) and reduces HDL's cardioprotective effects.

### 5.2 Impact of comorbidities: chronic inflammation

While patients with RA and SLE often present with HDL levels within the normal range, assessing alterations in HDL's specific components can be challenging. This difficulty arises because the majority of HDL particles are typically H1 (Table 1). However, it's noteworthy that dysfunctional HDL, such as H5, can be observed in both RA and SLE patients, indicating qualitative changes despite quantitative normalcy.

In RA, plasma HDL levels are often significantly altered with total HDL-C frequently reduced compared to healthy controls. A study reported that plasma H5% was notably higher in RA patients (median 7.2%, IQR 4.5%–8.9%) (18). Additionally, it was found that the level of Lp(a) serves as a significant predictor for high H5%. These findings suggest that H5 is involved in RA-related atherosclerosis and that long-term monitoring of plasma H5% may aid in evaluating ASCVD risk in RA, although further studies are needed to confirm this potential.

In SLE, alterations in HDL, including reductions in total HDL-C, and its immunomodulatory effects play a critical role in disease progression and clinical manifestations. Research by Gaal et al. has highlighted how changes in both the quantity and function of HDL are associated with SLE disease activity (61). Mechanisms contributing to these alterations include modifications in enzyme behavior, such as a decrease in PON1 activity, enhanced CETP activity, and the activation of remodeling enzymes (22). Autoantibodies targeting HDL and its components, especially ApoA-I, are sometimes found in SLE patients and further reduce HDL levels and impair its function. These alterations in HDL contribute to both disease activity and an increased risk of CVD, a frequent comorbidity in SLE (22).

### 5.3 Hormonal, modifiable, age-related, and sex-related influences

Modifiable lifestyle factors exert substantial effects on HDL level. Moderate alcohol intake is strongly associated with higher HDL-C, increasing levels by 9.0–13.1 mg/dL compared to non-drinkers (62). Obesity inversely correlates with HDL-C, while weight loss raises it by approximately 0.35 mg/dL per kilogram lost, likely through improved overall lipid metabolism (51). Regular exercise modestly boosts HDL-C by 3.0–3.3 mg/dL, thereby enhancing RCT (62). Smoking reduces HDL-C by 9.9 mg/dL in women and 2.6 mg/dL in men, exacerbating cardiovascular risk (63). High saturated fat intake impairs HDL function, whereas diets rich in unsaturated fats and fiber may improve HDL quality (64). Demographic factors also significantly influence HDL-C levels. It tends to decline with advancing age, and premenopausal women generally exhibit higher levels than men, largely attributable to the cardioprotective effects of estrogen (65). Additionally, as mentioned earlier, comorbid conditions, such as diabetes, liver disease, and chronic infections like sepsis can disrupt HDL metabolism (66). These disruptions often result in the formation of dysfunctional HDL particles, which may paradoxically elevate cardiovascular risk even when HDL-C levels appear normal. These various factors have complex interactions, often compounding risks in autoimmune patients. For instance, obesity, smoking, and elevated Lp(a) levels not only lower HDL-C but also correlate with higher H5%, amplifying systemic inflammation and further impairing HDL's anti-inflammatory properties (6, 18).

### 5.4 Pharmacologic modulators

Agents such as statins, fibrates, niacin, and corticosteroids exert distinct effects on HDL-C levels and functionality. Statins modestly increase HDL-C (5%–10%) by upregulating ApoA-I synthesis and reducing CETP activity, though their impact on HDL function remains debated (51, 67). Fibrates, which are PPAR-α agonists, increase HDL-C levels by 10%–20% primarily by promoting HDL production and enhancing LPL-mediated TG clearance, particularly in individuals with hypertriglyceridemia (68). Niacin (vitamin B3) provides the most robust HDL-C elevation (15%–35%), by inhibiting hepatic HDL catabolism and reducing CETP-mediated HDL remodeling, yet recent trials question its cardiovascular benefit (69). Corticosteroids paradoxically raise HDL-C but impair HDL's anti-inflammatory and CEC, potentially explaining their pro-atherogenic effects (70). While these agents can alter HDL quantity, therapies that reliably restore HDL function or improve dysfunctional HDL are currently limited. This highlights the need for treatments targeting HDL quality, such as CETP inhibitors or ApoA-I mimetics, which aim to improve HDL functionality rather than just raising HDL-C levels.

## 6 Clinical implications of dysfunctional HDL in CVD

### 6.1 Paradigm shift: function over concentration

From a clinical perspective, recent research highlights that HDL's cardioprotective efficacy is driven primarily by its functional quality rather than its abundance (11). This realization stems from the observation that HDL can become dysfunctional, losing its ability to promote RCT, exert anti-inflammatory effects, and protect endothelial function-even when present in high amounts (6). For example, oxidative modifications of HDL or its main protein component, ApoA-1, can impair its beneficial activities. Genetic studies have also shown that certain mutations can result in high HDL-C but paradoxically increase coronary event risk, further undermining the notion that “more is better” (6).

Clinical trials have reinforced this paradigm shift. Medications designed to raise HDL-C, such as CETP inhibitors and niacin, have failed to demonstrate reductions in cardiovascular events, despite significant increases in HDL-C levels (71, 72). These failures have prompted the development of new functional assays such as CEC and HDL inflammatory index that better predict cardiovascular outcomes than HDL-C concentration alone (5). As a result, current research and therapeutic strategies are increasingly focused on restoring or enhancing HDL functionality, through approaches like ApoA-1 mimetic peptides, reconstituted HDL infusions, and gene therapies targeting HDL metabolism (6). This shift in focus from concentration to function represents a crucial advance in the fight against residual cardiovascular risk.

HDL particles enriched with ApoC-III have been directly linked to increased CVD risk, highlighting the importance of particle composition in determining HDL's effects (73). ApoC-III can be incorporated into HDL during its secretion from the liver or intestine or transferred from VLDL in circulation. Once on HDL, ApoC-III was found to induce proinflammatory and pro-atherogenic effects on cells involved in atherosclerosis development (74, 75). Recent research has revealed that simply increasing HDL-C levels may not only be less beneficial than previously thought but could even be harmful in certain contexts. One reason is that interventions or genetic variants that raise HDL-C can also increase levels of ApoC-III, a protein known to inhibit LPL and impair TG metabolism. Some studies have shown that HDL particles enriched with ApoC-III lose their anti-atherogenic properties and may even promote vascular inflammation and plaque formation (76). This finding helps explain why clinical trials that focused solely on raising HDL-C, without considering the functional quality of HDL or its protein composition, failed to reduce cardiovascular events and, in some cases, may have inadvertently increased risk.

These alterations in HDL composition have broader implications, particularly when HDL shifts from protective to potentially harmful (Figure 1). This emphasizes the need to consider not only HDL-C levels but also the specific composition of HDL particles, particularly in the context of type 2 diabetes, where impaired insulin sensitivity and glucose uptake exacerbate cardiovascular risk (77). Despite these complexities, HDL-C remains a core variable in cardiovascular risk prediction models. Recent guidelines from the American Heart Association (AHA) and European Society of Cardiology (ESC) include HDL-C as a key parameter in their risk calculators (29, 78), which are widely applied in both clinical decision-making and population-based studies. Thus, regardless of the nature of its relationship with CVD, the use of HDL-C as a risk predictor remains unchallenged (3).

### 6.2 Role of electronegative HDL H5 in atherogenesis

This complexity is further illustrated by electronegative HDL H5, which has been implicated in the development of ASCVD and inflammatory conditions, particularly in autoimmune diseases like RA (18). Due to its electronegative properties, H5 exhibits impaired cholesterol efflux and diminished anti-inflammatory and antioxidative functions, promoting macrophage lipid accumulation and foam cell formation (18). It can also stimulate IL-6 and TNF-α secretion from endothelial cells and monocytes when combined with electronegative LDL, and it reduces cell number and viability, thereby contributing to early endothelial dysfunction and atherogenesis (79). Similarly, HDL dysfunction is observed in Alzheimer's disease, where oxidative modifications of ApoA-I and ApoE alter lipid metabolism and contribute to neuroinflammation (28). Given the shared pathways between neurodegenerative and CVD, observational evidence suggests that H5's dysfunction may play a broader role in systemic inflammation (Table 1) and lipid dysregulation (29).

### 6.3 Biomarker potential and prognostic value

Dysfunctional HDL has emerged as a significant biomarker in CVD, offering both diagnostic and prognostic value. Studies have shown that patients with CAD or heart failure often exhibit HDL with reduced functionality, independent of HDL-C levels (80). This dysfunction is linked to oxidative modifications, altered protein composition, and pro-inflammatory changes, making it a more reliable indicator of CVD risk than HDL-C alone. Prognostically, dysfunctional HDL is associated with adverse cardiovascular outcomes, including myocardial infarction and major adverse cardiac events (MACE). For instance, reduced CEC has been independently correlated with increased atherosclerotic burden and future cardiovascular events (5). Additionally, HDL's anti-inflammatory properties, when compromised, predict higher mortality in heart failure patients (81). These findings highlight the potential of dysfunctional HDL as a tool for risk stratification and personalized therapeutic strategies in CVD management.

## 7 Therapeutic strategies targeting HDL function

### 7.1 Limitations of HDL-C-raising therapies and novel agents enhancing HDL functionality

Nowadays, HDL functionality has become a key therapeutic target in CVD. Drugs such as CETP inhibitors (e.g., torcetrapib, dalcetrapib, and evacetrapib) effectively increased HDL-C levels but did not reduce cardiovascular events in large, randomized trials (82, 83). Indeed, some HDL-raising therapies may even generate dysfunctional HDL particles that lack anti-inflammatory or CEC, potentially explaining their lack of benefit (6).

Emerging agents aim to improve HDL's atheroprotective functions, including CEC, anti-inflammatory properties, and endothelial repair. Among the most promising approaches are ApoA-I mimetics, such as MDCO-216 and CER-001, which mimic the structure and function of nascent HDL to promote RCT (84, 85). Although early trials showed mixed results in plaque regression, these compounds demonstrate potential in stabilizing vulnerable plaques by reducing lipid content and inflammation (86).

Reconstituted HDL (rHDL) infusions, containing either wild-type or engineered ApoA-I variants and designed to boost RCT and reduce plaque inflammation (87), have demonstrated efficacy in preclinical models by improving both RCT and endothelial function (88). However, challenges remain, including variability in patient responses and the complexity of HDL's biological roles. These limitations underscore the need for novel biomarkers of HDL function and more targeted therapeutic approaches to translate HDL biology into effective CVD treatments. Another strategy involves small-molecule enhancers of HDL function as RVX-208 (now called apabetalone), which selectively upregulates ApoA-I production and improves HDL's anti-inflammatory properties (89–91).

RNA-based therapies, including antisense oligonucleotides (ASOs) and siRNA targeting ApoC-III, represent emerging biologics that modulate HDL function by enhancing CEC and reducing pro-atherogenic lipoproteins, thereby improving HDL quality beyond simply raising HDL-C levels (92).

Despite these advances, challenges remain, including patient heterogeneity and the need for reliable biomarkers to assess HDL functionality. Future therapies may combine functional HDL modulation with conventional lipid-lowering strategies to optimize cardiovascular outcomes.

### 7.2 Lifestyle and dietary interventions

Lifestyle and dietary interventions play a pivotal role in enhancing the functional properties of HDL, thereby contributing to cardiovascular health. Regular physical activity, particularly aerobic exercise, has been shown to improve HDL's antioxidative and anti-inflammatory functions. While evidence regarding exercise's impact on HDL's CEC is mixed, some studies suggest that exceeding a certain threshold of exercise intensity and duration may yield beneficial effects (93, 94).

Dietary patterns rich in antioxidants and healthy fats also positively influence HDL functionality. The Mediterranean diet, characterized by high consumption of fruits, vegetables, whole grains, legumes, nuts, and olive oil, has been associated with improvements in HDL's CEC, antioxidant properties, and anti-inflammatory functions (95). Specifically, components such as extra virgin olive oil, nuts, and fatty fish contribute to these enhancements (96). Additionally, moderate alcohol consumption, particularly red wine, may further augment HDL function due to the presence of polyphenols (97).

## 8 Future directions and research priorities

### 8.1 Gaps in current understanding

Future research should center on unraveling the mechanisms that govern HDL functionality, as current data reveal that its functional integrity outweighs mere HDL-C levels in predicting vascular benefit (78). Evidence indicates that pharmacologically raising HDL-C levels alone does not necessarily lead to better cardiovascular outcomes, making it essential to assess HDL functionality when evaluating cardiovascular risk (98). It is now evident that HDL particles differ in their protective abilities; notably, therapies that increase the production of small, atheroprotective HDL particles show particular promise. In patients with conditions such as RA, Janus kinase inhibitors (JAKi) have shown potential in not only restoring protective HDL functions but also increasing levels of these small-sized HDL particles. A study noted an inverse correlation between CRP levels and small-sized HDL levels, suggesting that reducing inflammation could improve HDL function and its associated atheroprotective effects (78). Thus, JAKi therapies may serve as a model for developing future therapeutics aimed at modifying HDL function.

Another growing area of research focuses on ApoA-I-based therapies (99). These therapies aim to directly enhance HDL functionality by increasing the activity of ApoA-I that is involved in cholesterol efflux. The variable efficacy of HDL-raising strategies, whether through lifestyle modification, pharmacological intervention, or genetic approaches, underscores the need to prioritize functional HDL assessment in both research and clinical trial design. Statin therapy has also been shown to affect HDL functionality (6). Specifically, statins appear to enhance cholesterol efflux from hepatoma cells, although their effects on macrophage cholesterol efflux are more variable. This suggests that future research should explore how statin therapy can be optimized to not only lower LDL but also improve the functional properties of HDL, particularly role in reducing atherosclerotic plaque formation.

### 8.2 Need for standardized functional assays

The functional properties of HDL are now seen as more relevant to cardiovascular protection than HDL-C content, but a major barrier to clinical application is the lack of standardized, validated assays for HDL function. Existing functional assays, including CEC, MPO and PON1 activity, and antioxidant capacity, all have notable limitations. For instance, while CEC is considered the gold standard for assessing HDL function and is inversely associated with CAD, it is labor-intensive, low-throughput, and requires cultured cells, making it impractical for routine clinical use. Other assays, such as those measuring cholesterol uptake capacity (CUC) or antioxidant activity, offer higher throughput and automation but still lack validation in large-scale clinical trials and standardization across laboratories (100, 101).

Recent technological advances that do not require cells for monitoring the CEC of HDL (102), like the HDL-SPE assay (103) and the automated CUC immunoassay (101), represent promising steps towards a more practical and reproducible functional testing. The diversity of available techniques, from ultracentrifugation and electrophoresis to NMR spectroscopy and direct immunoassays, highlights the complexity of HDL as a biomarker and the urgent need for consensus on which assay or combination of assays best reflects clinically meaningful HDL function (104). Establishing standardized, high-throughput, and clinically validated functional assays is therefore a top research priority, as it would enable more accurate risk stratification and facilitate the development of therapies targeting HDL functionality (100).

### 8.3 Integrative approaches: -omics, systems biology, and precision medicine

The future of HDL research and cardiovascular risk assessment is increasingly moving toward integrative approaches that leverage -omics technologies, systems biology, and precision medicine. High-throughput omics-including genomics, proteomics, lipidomics, and metabolomics allow for comprehensive profiling of HDL particles, revealing not only their cholesterol content but also their diverse protein and lipid cargo, post-translational modifications, and functional heterogeneity (105–107). These technologies have uncovered distinct HDL subpopulations with unique functional properties, some of which are more closely associated with cardiovascular protection or risk than total HDL-C levels. For instance, proteomic analyses have identified specific proteins such as PON1 and ApoC-III that can modulate HDL's anti-inflammatory and antioxidant capacities, providing new biomarkers and therapeutic targets (50).

Dysfunctional HDL is increasingly recognized as clinically important compared to LDL-, chylomicron-, and VLDL-focused approaches, which mainly address lipid levels, because HDL is multifunctional and its protective activities, cholesterol removal, antioxidant effects, support of endothelial health, and control of inflammation, cannot be replaced or captured by LDL, CM, or VLDL. Loss of these functions contributes to residual cardiovascular risk even when LDL is well controlled. System biology approaches, such as integrated proteomics and lipidomics, can reveal how dysfunctional HDL uniquely affects endothelial cells, immune responses, and plaque biology, identify factors driving HDL dysfunction, and pinpoint patient groups most at risk. This holistic perspective enables the identification of key molecular pathways and regulatory networks that govern HDL functionality and its interaction with other metabolic processes. Precision medicine, in turn, applies these insights to tailor prevention and treatment strategies to individual patients based on their unique genetic, molecular, and phenotypic profiles (105). By moving beyond population averages and simplistic biomarkers, these integrative strategies hold the promise of more accurately predicting cardiovascular risk and developing targeted interventions that restore or enhance HDL function in a personalized manner. As research advances, collaboration between clinicians, biologists, and data scientists will be essential to translating these discoveries into clinical practice. Together, these strategies provide mechanistic and translational insight, supporting the idea that restoring HDL function could complement traditional lipid-lowering therapies in preventing atherosclerosis and CVD.

## 9 Conclusion

It is increasingly apparent that not all HDL particles are the same, and that the quality of HDL is just as crucial as its quantity. This update on HDL dysfunction highlights the limitations of relying solely on HDL-C levels and underscores the growing value of incorporating HDL functional assessments into the broader, multifactorial evaluation of cardiovascular risk. As research advances, the focus is shifting towards understanding the mechanisms behind HDL dysfunction, particularly its altered composition and impaired ability to promote cholesterol efflux and protect the vasculature. Incorporating assessments of HDL quality, including dysfunction in subclasses like H5, alongside traditional measures will be critical for advancing heart disease prevention and treatment, enabling more personalized and effective strategies that focus on restoring HDL function rather than merely increasing HDL-C levels. By continuing to refine and update our understanding of HDL biology, researchers and clinicians can move beyond the traditional view of “good cholesterol” (108) toward a more functional and mechanistic perspective, unlocking new pathways for therapeutic innovation and improved patient outcomes.
